# Development of Mathematical Models Using circRNA Combinations (*circTulp4*, *circSlc8a1*, and *circStrn3*) in Mouse Brain Tissue for Postmortem Interval Estimation

**DOI:** 10.3390/ijms26104495

**Published:** 2025-05-08

**Authors:** Binghui Song, Jiewen Fu, Jie Qian, Ting He, Jingliang Cheng, Sawitree Chiampanichayakul, Songyot Anuchapreeda, Junjiang Fu

**Affiliations:** 1 Department of Medical Technology, Faculty of Associated Medical Sciences, Chiang Mai University, Chiang Mai 50200, Thailand; songbinghui@stu.swmu.edu.cn (B.S.); sawitree.chiampa@cmu.ac.th (S.C.); 2 Key Laboratory of Epigenetics and Oncology, The Research Center for Preclinical Medicine, Southwest Medical University, Luzhou 646000, China; fujiewen@swmu.edu.cn (J.F.); 20210199120039@stu.swmu.edu.cn (J.Q.); 20220199120039@stu.swmu.edu.cn (T.H.); jingliangc@swmu.edu.cn (J.C.); 3 Laboratory of Precision Medicine and DNA Forensic Medicine, The Research Center for Preclinical Medicine, Southwest Medical University, Luzhou 646000, China; 4 Laboratory of Forensic DNA, The Judicial Authentication Center, Southwest Medical University, Luzhou 646000, China; 5 Cancer Research Unit of Associated Medical Sciences (AMS CRU), Faculty of Associated Medical Sciences, Chiang Mai University, Chiang Mai 50200, Thailand; 6 Center of Excellence in Pharmaceutical Nanotechnology, Chiang Mai University, Chiang Mai 50200, Thailand

**Keywords:** postmortem interval (PMI), circular RNA (circRNA), forensic pathology, forensic genetics, forensic biomarkers, PMI estimation

## Abstract

The postmortem interval (PMI) is defined as the time interval between physiological death and the examination of the corpse, playing a critical role in forensic investigations. Traditional PMI estimation methods are often influenced by subjective and environmental factors. Circular RNAs (circRNAs), known for their stability, abundance, and conservation in brain tissue, show promise as biomarkers for PMI estimation. However, research on circRNAs in this context remains limited. This study aimed to develop PMI estimation models using circRNAs across multiple temperatures. By employing semi-quantitative reverse transcription-PCR, *circTulp4*, *circSlc8a1*, and *circStrn3* were identified as reliable biomarkers for mouse brain tissue. Mathematical models were constructed using the reference genes *28S* rRNA, *mt-co1*, and *circCDR1as*. At 4 °C, most equations had *p*-values below 0.05, with the equation using *circSlc8a1* as a marker exhibiting the highest goodness of fit. Validation results indicated that the equation using *circTulp4* as the reference gene had the highest accuracy. When applying the combined aforementioned three circRNAs, the equation using *circCDR1as* as the reference gene showed better accuracy. At 25 °C, all equations had R^2^ values greater than 0.86, but most cubic equations had *p*-values above 0.05. Validation results demonstrated that the *circTulp4*/*mt-co1* equation had the highest accuracy. When applying combined circRNAs, the R^2^ values improved, and long-term PMI estimation was more accurate than short-term PMI estimation. At 35 °C, the linear equations had significantly poorer goodness of fit compared to nonlinear equations, and nonlinear equations exhibited better accuracy than linear equations. When applying the combined aforementioned three circRNAs, the accuracy of the three reference genes was similar, and the accuracy of long-term PMI estimation was consistently higher than that of short-term estimation. For the three-dimensional models, all R^2^ values exceeded 0.75 with *p*-values significantly below 0.0001. Validation results demonstrated higher accuracy at 25 °C and 35 °C, with superior performance for long-term PMI estimation. In summary, this study constructed PMI estimation models under multiple temperature conditions based on highly expressed circRNAs in mouse brain tissue, highlighting *circTulp4*, *circSlc8a1*, and *circStrn3* as novel biomarkers. These findings offer a complementary tool for PMI estimation, particularly for long-term PMI estimation.

## 1. Introduction

The postmortem interval (PMI) refers to the time that has elapsed since an individual’s death. It plays a vital role in forensic investigations, as estimating the time of death provides significant insights into determining the time of the crime, defining the investigation scope, and verifying alibis [1]. After death, the human body undergoes a series of physical and chemical changes that lead to tissue decomposition [2,3]. Postmortem changes are inevitable and gradual, typically categorized into early postmortem changes (within 24 h after death) and late postmortem changes. Several variables influence the progression of the postmortem period, including environmental conditions, human factors, and individual characteristics, making it difficult to infer the time of death [4,5]. Most early research on PMI estimation focused on the initial stages, from death until the body reached ambient temperature. However, once decomposition begins, PMI estimation becomes increasingly difficult and less accurate. PMI estimation has long been a critical issue in forensic identification. Most traditional methods are qualitative and descriptive, and they still face many challenges. Research indicates that more accurate PMI estimates require quantifiable methods [6]. With advancements in biochemistry and molecular biology, molecular markers such as DNA, RNA, and proteins have emerged as novel tools for PMI estimation [7,8,9]. Among these, various RNAs, including messenger RNA (mRNA), ribosomal RNA (rRNA), and microRNA (miRNA), have been investigated for PMI estimation.

Circular RNA (circRNA) was first identified in 1976 [10] and is classified as a type of non-coding RNA (ncRNA) with a covalently closed circular structure. Unlike linear RNA, circRNA lacks both a 5′-end cap and a 3′-end poly(A) tail, making it resistant to degradation by Ribonuclease R (RNase R) [11,12,13]. It is predominantly found in exosomes and the cytoplasm [14,15] and exhibits tissue, cell-type, and developmental-stage specificity [16,17]. Compared to typical nucleic acids, circRNA’s circular configuration grants it a longer half-life and stable expression [18], making it a promising candidate for forensic applications. Functionally, circRNAs act as miRNA sponges through the competitive endogenous RNA (ceRNA) mechanism [19]. By binding to miRNAs, circRNAs inhibit their function and alleviate their suppressive effects on target genes, thereby promoting gene expression. Additionally, circRNAs regulate transcription, encode proteins, participate in translation, and modulate the cell cycle and apoptosis [20], and they have been extensively studied in areas such as physiology, cancer, immune responses, cardiovascular health, metabolic diseases, and vaccines [21,22,23,24].

Recently, researchers have explored the forensic potential of circRNA, including its use in estimating an individual’s age, identifying tissues and body fluids, determining the formation time of bloodstains, and analyzing causes of death. The inherent stability, abundance, and tissue specificity of circRNA highlight its potential in PMI estimation. However, few studies have focused on developing circRNA-based PMI estimation models. Research shows that circRNAs exhibit the highest expression and specificity in brain tissue compared to other organs [25]. Moreover, circRNAs are conserved between humans and mice, with many highly expressed circRNAs in mice also being prevalent in humans [26]. The brain, being anatomically enclosed, is less exposed to environmental factors compared to tissues like the skin, which are more vulnerable to external influences. This relative isolation suggests that brain tissue, particularly its circRNA profile, may be more closely linked to PMI, especially under controlled temperature conditions. Therefore, circRNAs in brain tissue hold great promise as biomarkers for PMI estimation.

This study examines the postmortem degradation patterns of circRNAs across different PMIs and temperature conditions to identify reliable circRNA biomarkers and develop PMI estimation models. Focusing on brain tissue, samples were collected at various PMIs under different temperatures, including low (4 °C), room (25 °C), and high (35 °C) temperatures. Homologous circRNAs in mice were identified, and specific primers were designed for their detection. To evaluate postmortem changes, the semi-quantitative reverse transcription polymerase chain reaction (RT-PCR) was performed. Based on the findings, circRNA-based PMI estimation models were developed for different temperature conditions and subsequently validated for accuracy. The successful establishment of these mathematical models based on circRNAs provides a novel and valuable tool for PMI estimation, enhancing their forensic application.

## 2. Results

**Figure 1:**
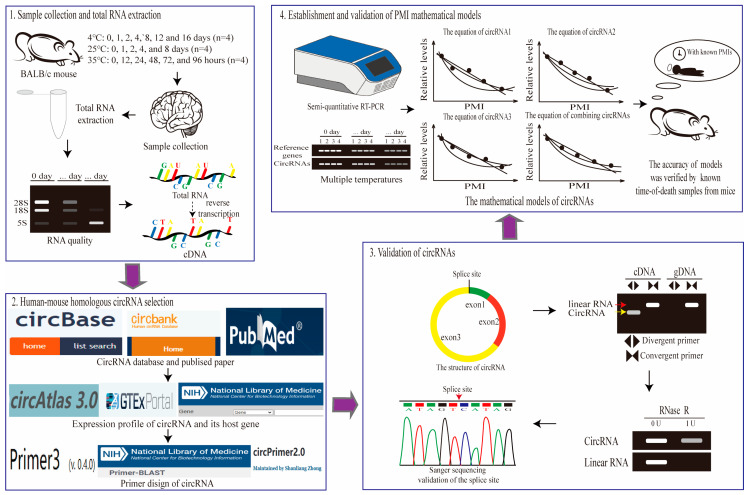
The experimental and analytical workflow in this study. To construct PMI estimation models based on circRNAs at multiple temperatures, the following workflow will be performed. This workflow consists of four main parts: sample collection and total RNA extraction, human–mouse homologous circRNAs selection, validation of circRNAs, and establishment and validation of PMI estimation models.

To facilitate the development of the PMI estimation models, the experimental and analytical workflow of this study is illustrated in Figure 1.

### 2.1. The Selection of Human–Mouse Homologous circRNA

**Figure 2:**
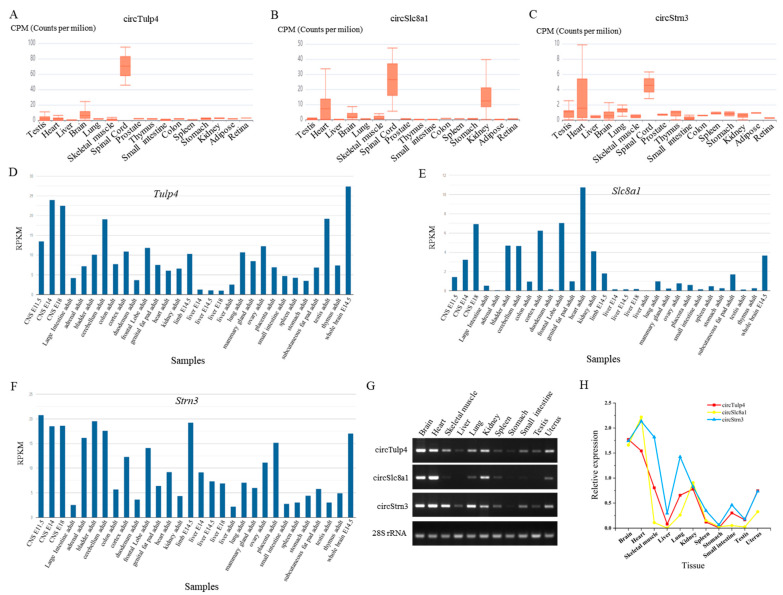
The expression of circRNAs and their host genes in different mouse tissues. (**A**–**C**) The expression of *circTulp4* (mmu_circ_0000723), *circSlc8a1* (mmu_circ_0000823), and *circStrn3* (mmu_circ_0000372) in multiple tissues based on the circAtlas database. (**D**–**F**) The expression of *Tulp4*, *Slc8a1*, and *Strn3* genes in multiple tissues based on the NCBI database. (**G**) The agarose gel electrophoresis results of *circTulp4*, *circSlc8a1*, *circStrn3*, and *28S* rRNA in 11 different tissues. (**H**) The relative expression of *circTulp4*, *circSlc8a1*, and *circStrn3* in 11 different tissues.

According to the literature and circRNA databases, circRNAs that were highly expressed and conserved in brain tissue were selected. Based on the pre-experiment research, three circular transcripts were screened as candidate biomarkers, including *circTulp4* (mmu_circ_0000723), *circSlc8a1* (mmu_circ_0000823), and *circStrn3* (mmu_circ_0000372). Notably, they were expressed in multiple tissues of mice and showed relatively high expression in brain tissue (Figure 2A–C). The expression of their host genes was queried in the NCBI database, revealing that *Tulp4*, *Slc8a1*, and *Strn3* were highly expressed in brain tissue (Figure 2D–F). Agarose gel electrophoresis results from 11 mouse tissues indicated that these three circRNAs were highly expressed in brain tissue and showed notable expression in other tissues, including the heart, skeletal muscle, lung, and kidney (Figure 2G). Quantitative analysis results clearly demonstrated that these circRNAs were highly expressed in mouse brain tissue (Figure 2H). Additionally, the expression of homologous human circRNAs and their host genes was examined in the circBank and GTEx databases. The results showed that *circTulp4*, *circSlc8a1*, and *circStrn3* exhibited relatively high expression in human brain tissue (Supplementary Figure S1A,C,E). Based on the sequences of homologous circRNAs in humans and mice, homology analysis results indicated that *circTulp4*, *circSlc8a1*, and *circStrn3* shared 80%, 88%, and 91% sequence similarity, respectively (Supplementary Figure S1B,D,F). GTEx data further demonstrated that the host genes were expressed across multiple regions of the brain tissue (Supplementary Figure S2A–C).

### 2.2. Validation of circRNAs

**Figure 3:**
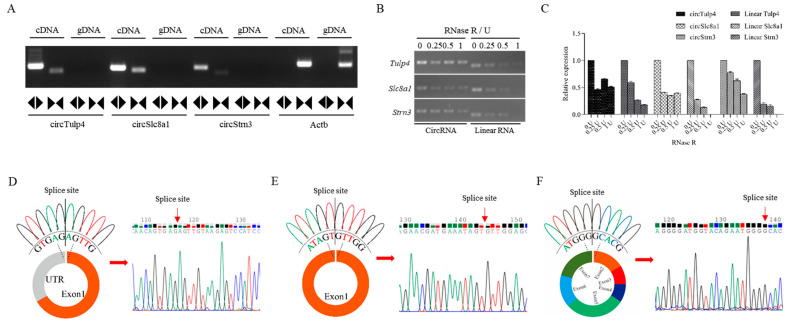
Validation of *circTulp4*, *circSlc8a1*, and *circStrn3* in mouse brain tissue. (**A**) The electrophoresis analysis of convergent and divergent primers of *circTulp4*, *circSlc8a1*, *circStrn3*, and *Actb* in cDNA and gDNA samples. (**B**) The RNase R digestion analysis of circRNAs and their corresponding linear RNAs in 0 U, 0.25 U, 0.5 U, and 1 U groups. (**C**) The data analysis of the relative expression of circRNAs and their corresponding linear RNAs after the RNase R digestion. (**D**–**F**) The circular structure of *circTulp4*, *circSlc8a1*, and *circStrn3* and the results of Sanger sequencing. Colored peaks represent individual bases in the sequencing chromatogram: green = A, red = T, blue = C, and black = G.

Primer testing can validate the structure of circRNAs by using divergent primers for circRNA amplification and convergent primers for linear RNA amplification. Agarose gel electrophoresis results demonstrated that *Tulp4*, *Slc8a1*, and *Strn3* in brain tissue cDNA were detected by both divergent and convergent primers, yielding bands of the expected size (Figure 3A). However, in gDNA samples, divergent primers failed to amplify the corresponding circRNA bands. Since the convergent primers for these corresponding linear RNAs span introns, they did not produce detectable bands in gDNA samples. As a control, *Actb* was only amplified by convergent primers in both cDNA and gDNA. After digesting total RNA with different concentrations of RNase R, cDNA was synthesized through reverse transcription. At high concentrations of RNase R digestion (one active unit (U)), circRNAs remained detectable with clear bands, whereas their corresponding linear RNAs were completely degraded (Figure 3B). Relative quantitative analysis further confirmed the greater resistance of circRNAs to RNase R digestion (Figure 3C). Based on database prediction and circPrimer 2.0, the structures, types, and splicing sites of the three circRNAs were identified. Sanger sequencing validated their splice sites, which matched computational predictions, confirming that they are circular sequences formed by back-splicing (Figure 3D–F).

### 2.3. Construction and Validation of PMI Estimation Models by circRNAs at Low Temperature

**Figure 4:**
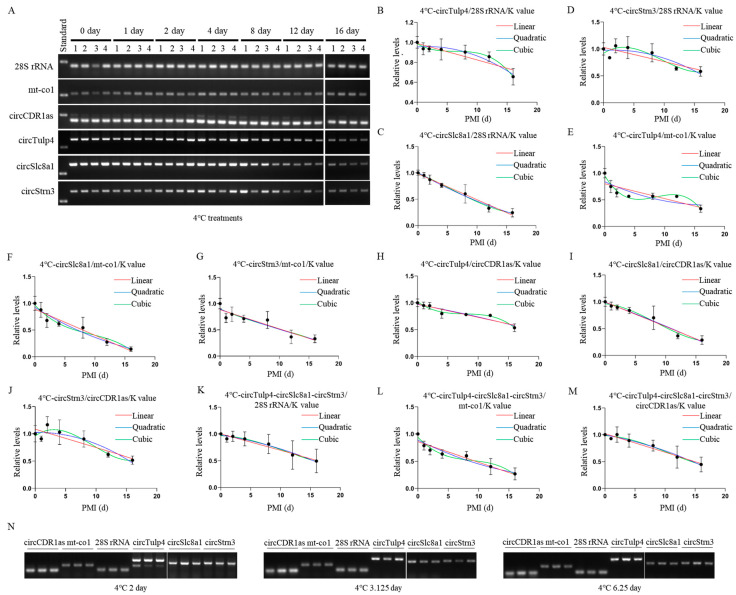
Construction and validation of 4 °C PMI estimation models. (**A**) The postmortem levels of reference genes *28S* rRNA, *mt-co1*, *circCDR1as* and the biomarkers *circTulp4*, *circSlc8a1*, and *circStrn3* in brain tissue on days 0, 1, 2, 4, 8, 12, and 16 at 4 °C. (**B**–**D**) The linear, quadratic, and cubic equations constructed at 4 °C based on circTulp4 and the three reference genes *28S* rRNA, *mt-co1*, and *circCDR1as*, respectively. (**E**–**G**) The linear, quadratic, and cubic equations constructed at 4 °C based on circSlc8a1 and the three reference genes *28S* rRNA, *mt-co1*, and *circCDR1as*, respectively. (**H**–**J**) The linear, quadratic, and cubic equations constructed at 4 °C based on *circStrn3* and the three reference genes *28S* rRNA, *mt-co1*, and *circCDR1as*, respectively. (**K**–**M**) After the combination of circRNAs, the linear, quadratic, and cubic equations were constructed at 4 °C based on the three reference genes *28S* rRNA, *mt-co1*, and *circCDR1as*, respectively. (**N**) The electrophoresis results of the validation samples for the 4 °C equation at 2, 3.125, and 6.25 days.

The postmortem degradation levels of circRNAs and reference genes at 4 °C were analyzed using agarose gel electrophoresis. The results showed that reference gene degradation levels remained stable for up to 16 days postmortem, whereas circRNAs exhibited significant degradation from day 8 onward (Figure 4A). Mathematical models were successfully established based on grayscale values from semi-quantitative RT-PCR at 4 °C, with *28S* rRNA, cytochrome c oxidase subunit I (*mt-co1*), and *circCDR1as* as reference genes (Table 1 and Supplementary Table S1). In these models, the relative postmortem levels and PMI were used as the Y and X values of the equation, respectively, while the ratio of the marker’s grayscale values to the reference gene on day 0 was designated as a constant (K). The calculation formula is detailed in the methods section. After calculation, using *28S* rRNA as the reference gene, the K values for *circTulp4*, *circSlc8a1*, and *circStrn3* were 1.154, 1.228, and 0.8010, respectively. Using *mt-co1* as the reference gene, the K values were 3.905, 3.532, and 2.281, respectively. Using *circCDR1as* as the reference gene, the K values were 0.9492, 0.8539, and 0.5634, respectively.

For *circTulp4*, mathematical models were successfully constructed using *28S* rRNA, *mt-co1*, and *circCDR1as* as reference genes, and the results were visualized (Figure 4B–D). Among these, only the quadratic equation of *circTulp4*/*mt-co1* (0.0799) had a *p*-value greater than 0.05. All equations had an R^2^ value above 0.6, with cubic equations showing the best fit for each reference gene. Notably, the *circTulp4*/*circCDR1as* equation exhibited the highest fit.

For *circSlc8a1*, mathematical models were successfully constructed using *28S* rRNA, *mt-co1*, and *circCDR1as* as reference genes, and the results were visualized (Figure 4E–G). All equations had *p*-values below 0.05 and R^2^ values above 0.8, with the *circSlc8a1*/*28S* rRNA equation exhibiting the best fit.

For *circStrn3*, mathematical models were successfully constructed using *28S* rRNA, *mt-co1*, and *circCDR1as* as reference genes, and the results were visualized (Figure 4H–J). Most equations had *p*-values below 0.05, except for the cubic equations of *circStrn3*/*28S* rRNA (*p* = 0.0905) and *circStrn3*/*mt-co1* (*p* = 0.0768). All equations had R^2^ values above 0.5, with the equations using *mt-co1* as the reference gene exhibiting a better fit.

When combining the three circRNAs, mathematical models were successfully constructed using *28S* rRNA, *mt-co1*, and *circCDR1as* as reference genes, and the results were visualized (Figure 4K–M). All equations had *p*-values below 0.05 and R^2^ values above 0.6, with those using *mt-co1* as the reference gene exhibiting a better fit. However, no significant improvement in equation fit or statistical significance was observed compared to single biomarkers.

For the equations at 4 °C, the accuracy of PMI estimation was tested using samples with known PMIs for days 2, 3.125, and 6.25 (Figure 4N). In the equations constructed with *circTulp4* as the marker, the validation results indicated that when using *28S* rRNA and *circCDR1as* as reference genes, linear equations were more accurate than nonlinear ones. However, when using *mt-co1* as the reference gene, nonlinear equations were more accurate. Additionally, the *circTulp4*/*circCDR1as* models demonstrated higher accuracy. For *circSlc8a1*-based models, the validation results indicated that *circSlc8a1*/*28S* rRNA models provided higher accuracy. When using *28S* rRNA and *mt-co1* as reference genes, nonlinear equations were more accurate than linear ones, while equations using *circCDR1as* as the reference gene performed better in their linear form. For *circStrn3*-based models, the validation results indicated that *circStrn3*/*28S* rRNA demonstrated higher accuracy. In the equations using *28S* rRNA as the reference gene, linear equations were more accurate than nonlinear ones. Additionally, the day 3.125 samples showed lower accuracy across all models. In the equations constructed by combining the three circRNAs, the validation results indicated that when using *28S* rRNA and *circCDR1as* as reference genes, linear equations were more accurate than nonlinear ones. However, in the equations using *mt-co1* as the reference gene, nonlinear equations were more accurate. Moreover, the day 3.125 samples showed lower accuracy across all models, and long-term PMI estimation exhibited slightly better accuracy than short-term PMI estimation. Additional validation details are provided in Table 2 and Supplementary Table S2.

### 2.4. Construction and Validation of PMI Estimation Models by circRNAs at Room Temperature

**Figure 5:**
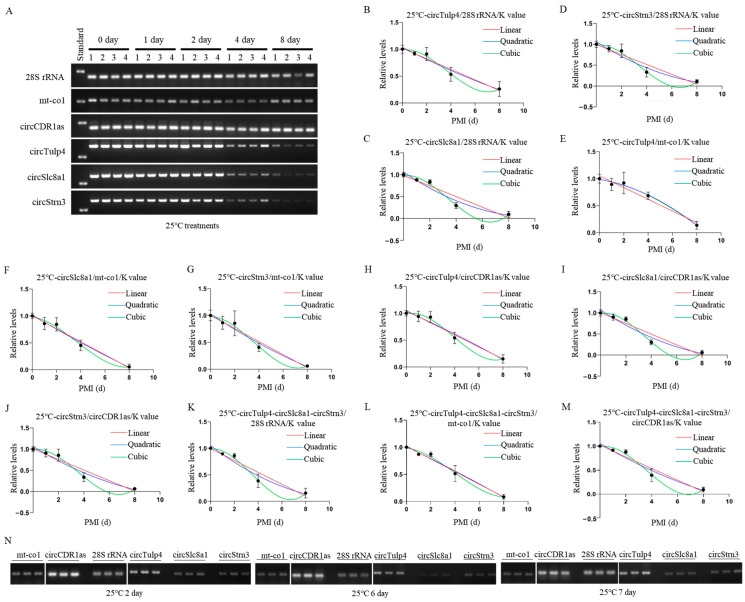
Construction and validation of 25 °C PMI estimation models. (**A**) The postmortem levels of reference genes *28S rRNA*, *mt-co1*, *circCDR1as* and the biomarkers *circTulp4*, *circSlc8a1*, and *circStrn3* in brain tissue on days 0, 1, 2, 4, and 8 at 25 °C. (**B**–**D**) The linear, quadratic, and cubic equations constructed at 25 °C based on *circTulp4* and the three reference genes *28S* rRNA, *mt-co1*, and *circCDR1as*, respectively. (**E**–**G**) The linear, quadratic, and cubic equations constructed at 25 °C based on *circSlc8a1* and the three reference genes *28S* rRNA, *mt-co1*, and *circCDR1as*, respectively. (**H**–**J**) The linear, quadratic, and cubic equations constructed at 25 °C based on circStrn3 and the three reference genes *28S* rRNA, *mt-co1*, and *circCDR1as*, respectively. (**K**–**M**) After the combination of circRNAs, the linear, quadratic, and cubic equations were constructed at 25 °C based on the three reference genes, *28S* rRNA, *mt-co1*, and *circCDR1as*, respectively. (**N**) The electrophoresis results of the validation samples for the 25 °C equation at 2, 6, and 7 days.

The postmortem degradation levels of circRNAs and reference genes at 25 °C were analyzed using agarose gel electrophoresis. The results showed that the degradation level of the reference gene remained stable for up to 8 days postmortem, while circRNAs exhibited significant degradation starting on day 2 postmortem (Figure 5A). Mathematical models were successfully established based on grayscale values from semi-quantitative RT-PCR at 25 °C, with *28S* rRNA, *mt-co1*, and *circCDR1as* as reference genes (Table 3 and Supplementary Table S3). After calculation, when using *28S* rRNA as the reference gene, the K values of the biomarkers *circTulp4*, *circSlc8a1*, and *circStrn3* were 1.320, 1.233, and 1.070, respectively. Using *mt-co1* as the reference gene, the K values were 1.950, 1.821, and 1.583, respectively. Using *circCDR1as* as the reference gene, the K values were 1.156, 1.082, and 0.937, respectively.

For *circTulp4*, mathematical models were successfully constructed using *28S* rRNA, *mt-co1*, and *circCDR1as* as reference genes, and the results were visualized (Figure 5B–D). In these mathematical models, all cubic equations had *p*-values above 0.05. All equations had an R^2^ value above 0.86. Each reference gene’s cubic equation demonstrated the highest fit, with the *circTulp4*/*circCDR1as* equation exhibiting the best fit.

For *circSlc8a1*, mathematical models were successfully constructed using *28S* rRNA, *mt-co1*, and *circCDR1as* as reference genes, and the results were visualized (Figure 5E–G). All linear equations had *p*-values below 0.05. Among the nonlinear equations, only the quadratic equation for *circSlc8a1*/*mt-co1* had a *p*-value below 0.05. The R^2^ values were all greater than 0.89, with the cubic equations for each reference gene showing the highest fit.

For *circStrn3*, mathematical models were successfully constructed using *28S* rRNA, *mt-co1*, and *circCDR1as* as reference genes, and the results were visualized (Figure 5H–J). All cubic equations had *p*-values above 0.05, and the quadratic equation for *circStrn3*/*28S* rRNA also had a *p*-value above 0.05. All equations had R^2^ values above 0.86, with the equations using *circCDR1as* as the reference gene showing a better fit.

When combining the three circRNAs, mathematical models were successfully constructed using *28S* rRNA, *mt-co1*, and *circCDR1as* as reference genes, and the results were visualized (Figure 5K–M). All cubic equations had *p*-values above 0.05, and the quadratic equation using *28S* rRNA as the reference gene also had a *p*-value greater than 0.05. All equations had R^2^ values above 0.9, with those using *mt-co1* as the reference gene showing a better fit. Compared to single markers, combining circRNAs improved the fit of the equations.

For the equations at 25 °C, the accuracy of PMI estimation was tested using samples with known PMIs for days 2, 6, and 7 (Figure 5N). In the equations constructed with *circTulp4* as the marker, *circTulp4*/*28S* rRNA and *circTulp4*/*mt-co1* showed higher accuracy in short-term PMI estimation. Moreover, the validation results indicated that *circTulp4*/*mt-co1* demonstrated higher accuracy. In the equations constructed with *circSlc8a1* as the marker, *circSlc8a1*/*28S* rRNA showed higher accuracy. The validation results indicated that nonlinear equations were more accurate for day 2 and 6 estimations, while linear equations were more accurate for day 7 estimation. The results also showed that *circSlc8a1* performed better in long-term PMI estimation. In the equations constructed with *circStrn3* as the marker, *circStrn3*/*28S* rRNA showed higher accuracy. The validation results indicated that nonlinear equations were more accurate for day 2 estimation, while linear equations were more accurate for day 7 estimation. Additionally, *circStrn3* performed better in long-term PMI estimation. In the equations constructed by combining the three circRNAs, the validation results showed that nonlinear equations were more accurate for day 2 samples, while linear equations were more accurate for day 7 samples. In the equations using *mt-co1* as the reference, nonlinear equations demonstrated higher accuracy. Moreover, the day 2 samples had lower accuracy across all models, and long-term PMI estimation was slightly more accurate than short-term PMI estimation. Additional validation details are provided in Table 4 and Supplementary Table S4.

### 2.5. Construction and Validation of PMI Estimation Models by circRNAs at High Temperature

**Figure 6:**
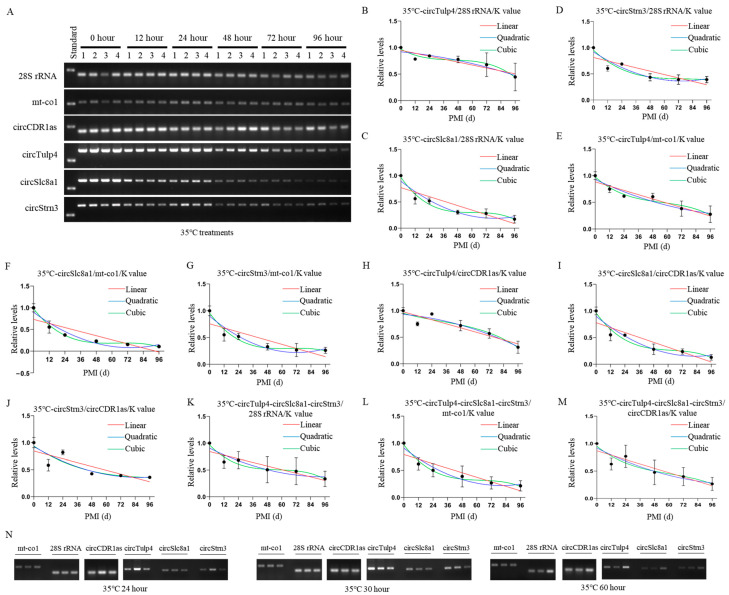
Construction and validation of 35 °C PMI estimation models. (**A**) The postmortem levels of reference genes *28S* rRNA, *mt-co1*, *circCDR1as* and the biomarkers *circTulp4*, *circSlc8a1*, and *circStrn3* in brain tissue on hours 0, 12, 24, 48, 72, and 96 at 35 °C. (**B**–**D**) The linear, quadratic, and cubic equations constructed at 35 °C based on circTulp4 and the three reference genes *28S* rRNA, *mt-co1*, and *circCDR1as*, respectively. (**E**–**G**) The linear, quadratic, and cubic equations constructed at 35 °C based on *circSlc8a1* and the three reference genes *28S* rRNA, *mt-co1*, and *circCDR1as*, respectively. (**H**–**J**) The linear, quadratic, and cubic equations constructed at 35 °C based on *circStrn3* and the three reference genes *28S* rRNA, *mt-co1*, and *circCDR1as*, respectively. (**K**–**M**) After the combination of circRNAs, the linear, quadratic, and cubic equations were constructed at 35 °C based on the three reference genes *28S* rRNA, *mt-co1*, and *circCDR1as*, respectively. (**N**) The electrophoresis results of the validation samples for the 35 °C equation at 24, 30, and 60 h.

The postmortem degradation levels of circRNAs and reference genes at 35 °C were analyzed using agarose gel electrophoresis. The results showed that the degradation level of the reference gene *mt-co1* remained stable for up to 72 h postmortem, while circRNAs exhibited significant degradation starting at 24 h postmortem (Figure 6A). Mathematical models were successfully established based on grayscale values from semi-quantitative RT-PCR at 35 °C, with *28S* rRNA, *mt-co1*, and *circCDR1as* as reference genes (Table 5 and Supplementary Table S5). After calculation, when using *28S* rRNA as the reference gene, the K values of the biomarkers *circTulp4*, *circSlc8a1*, and *circStrn3* were 1.196, 1.216, and 0.797, respectively. Using *mt-co1* as the reference gene, the K values were 3.879, 3.455, and 2.257, respectively. Using *circCDR1as* as the reference gene, the K values were 0.861, 0.771, and 0.574, respectively.

For *circTulp4*, mathematical models were successfully constructed using *28S* rRNA, *mt-co1*, and *circCDR1as* as reference genes, and the results were visualized (Figure 6B–D). In these equations, the *p*-values for all cubic equations were greater than 0.05. All equations had an R^2^ value above 0.55, and the cubic equations for each reference gene showed the highest fit, with the *circTulp4*/*mt-co1* equation exhibiting the best fit.

For *circSlc8a1*, mathematical models were successfully constructed using *28S* rRNA, *mt-co1*, and *circCDR1as* as reference genes, and the results were visualized (Figure 6E–G). Except for the cubic equation of *circSlc8a1*/*circCDR1as*, all equations had *p*-values below 0.05 and R^2^ values above 0.7.

For *circStrn3*, mathematical models were successfully constructed using *28S* rRNA, *mt-co1*, and *circCDR1as* as reference genes, and the results were visualized (Figure 6H–J). Some nonlinear equations had *p*-values above 0.05, and all equations had R^2^ values above 0.65, with the *circStrn3*/*circCDR1as* equation showing a poorer fit.

When combining the three circRNAs, mathematical models were successfully constructed using *28S* rRNA, *mt-co1*, and *circCDR1as* as reference genes and visualized (Figure 6K–M). In these equations, except for the cubic equations using *28S* rRNA and *circCDR1as* as reference genes, all *p*-values were less than 0.05. All equations had R^2^ values above 0.55, with those using *mt-co1* as the reference gene showing a better fit. However, compared to single markers, there was no significant improvement in equation fit or statistical significance.

For the equations at 35 °C, the accuracy of PMI estimation was tested using samples with known PMIs for hours 24, 30, and 60 (Figure 6N). In the equations using *circTulp4* as the marker, *circTulp4*/*28S* rRNA showed higher accuracy. The validation results indicated that quadratic equations were more accurate when *28S* rRNA was used as the reference gene. Additionally, long-term PMI estimation was slightly more accurate than short-term PMI estimation. Similarly, in equations using *circSlc8a1* as the marker, *circSlc8a1*/*circCDR1as* showed higher accuracy. The validation results indicated that nonlinear equations were more accurate than linear equations when *28S* rRNA and *mt-co1* were used as reference genes. Moreover, long-term PMI estimation was slightly more accurate than short-term PMI estimation. In the equations constructed with *circStrn3* as the marker, *circStrn3*/*circCDR1as* showed lower accuracy. The validation results indicated that nonlinear equations were more accurate than linear equations when *mt-co1* and *circCDR1as* were used as reference genes. Additionally, quadratic equations demonstrated higher accuracy in long-term PMI estimation. In the equations combining the three circRNAs, estimation errors were similar across the equations using the three reference genes. The validation results indicated that nonlinear equations were generally more accurate than linear equations. However, when using *mt-co1* as the reference gene, linear equations demonstrated higher accuracy. Moreover, long-term PMI estimation was slightly more accurate than short-term PMI estimation. Additional validation details are provided in Table 6 and Supplementary Table S6.

### 2.6. Construction and Validation of Three-Dimensional Models for PMI Estimation

**Figure 7:**
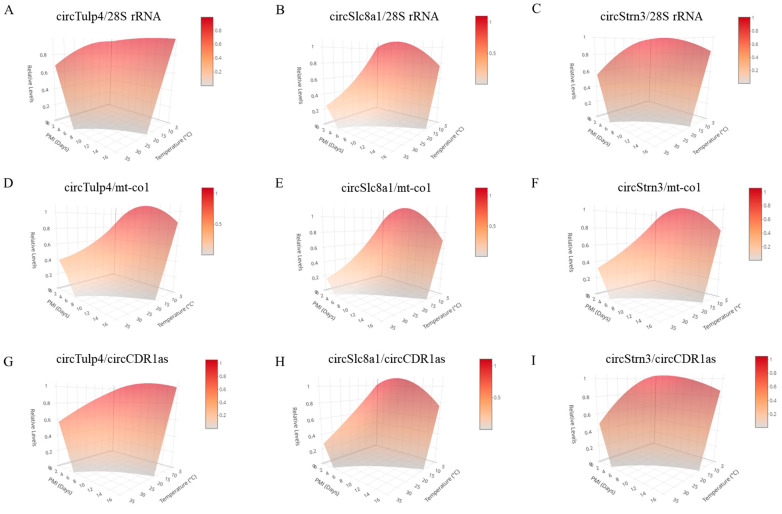
The three-dimensional models for PMI estimation were constructed based on data at 4 °C, 25 °C, and 35 °C. (**A**–**C**) These three-dimensional models were constructed using reference gene *28S* rRNA in combination with *circRNAs*, *circTulp4*, *circSlc8a1*, and *circStrn3*, respectively. (**D**–**F**) These three-dimensional models were constructed using reference gene *mt-co1* in combination with circRNAs, *circTulp4*, *circSlc8a1*, and *circStrn3*, respectively. (**G**–**I**) These three-dimensional models were constructed using reference gene *circCDR1as* in combination with circRNAs, *circTulp4*, *circSlc8a1*, and *circStrn3*, respectively. A color scale bar is included in the top right corner of the 3D plot to indicate the mapping between surface color and circRNA relative expression levels. Warmer colors (red) represent higher expression levels, while cooler tones (gray) correspond to lower expression levels.

To construct a model capable of inferring the PMI across multiple temperatures, three-dimensional estimation models were established based on the relative expression data at 4 °C, 25 °C, and 35 °C (Figure 7). The three-dimensional models were built by standardizing the PMI units to days across different temperatures. The results showed that these three-dimensional equations exhibit a high degree of fit, with *p*-values far less than 0.0001 (Table 7). In addition, the three-dimensional models were validated using validation samples collected under different temperature conditions. For *circTulp4*, the model demonstrated higher accuracy at 25 °C and 35 °C, especially for long-term PMI estimation. For *circSlc8a1*, under low-temperature conditions, the equation using *28S* rRNA as the reference gene performed best. Furthermore, at 25 °C and 35 °C, the model showed higher accuracy for long-term PMI estimation. For *circStrn3*, under low-temperature conditions, using *circCDR1as* as the reference gene yielded higher accuracy. Overall, in most environmental conditions, the model showed better accuracy for estimating longer PMIs. Additional validation details are provided in Supplementary Table S7. Since the three-dimensional models have relatively limited temperature conditions, incorporating more postmortem change data of circRNAs under additional temperature conditions, particularly low-temperature conditions, may improve the accuracy of these models.

## 3. Discussion

PMI is defined as the time interval between physiological death and the forensic examination of the corpse. Traditional methods for estimating PMI based on postmortem phenomena are often imprecise and heavily reliant on experience. For example, algor mortis becomes ineffective for late-stage PMI estimation because body temperature stabilizes once it reaches the ambient temperature. Additionally, environmental factors can significantly affect and damage the corpse in late-stage PMI estimation. With advancements in molecular biology techniques, researchers have explored biomarkers for PMI detection. Due to the abundance of RNases in the environment and within organisms, RNA generally degrades rapidly after death. However, early studies have suggested a potential correlation between RNA degradation and PMI. Bauer [27] highlighted the potential and practical applications of RNA technology in forensic science, revealing that various factors influence postmortem RNA stability, with RNA in brain tissue remaining relatively stable within 96 h after death. Multiparameter mathematical models applied to RNA analysis can estimate PMI with measurable error rates under various temperature conditions. However, these models still have limitations, particularly for late-stage PMI estimation [8].

In recent years, the rapid development of high-throughput sequencing technologies and computational pipelines has significantly advanced the discovery and research of circular RNAs (circRNAs). Due to their relatively stable structure and tissue-, cell-, and developmental stage-specific expression, circRNAs hold strong potential for PMI estimation, although related research remains limited. CircRNAs are endogenous biomolecules formed by the back-splicing of pre-mRNA into a covalently closed circular structure, functioning as miRNA sponges, participating in protein translation, regulating transcription, and playing important roles in physiology, cancer, and diseases. Current research indicates that circRNAs are most abundantly and specifically expressed in the human brain [25] and are highly enriched in the mammalian brain [28]. Additionally, studies have shown that circRNAs are highly concentrated in synaptic regions of neuronal compartments, and most circRNAs that are well expressed in mice are also highly expressed in humans [26]. Moreover, the brain is anatomically closed, providing a relatively stable intracranial environment that is less affected by external factors. As it decomposes later than other tissues after death, brain tissue serves as a more stable and reliable specimen for PMI estimation. Compared to more exposed tissues (e.g., skin), brain tissue degradation, when temperature is controlled as the primary influencing factor, may exhibit a stronger correlation with PMI.

This study, using a mouse model, identified three highly expressed circRNAs with human–mouse homology, including *circTulp4*, *circSlc8a1*, and *circStrn3*. *CircTulp4* (mmu_circ_0001746) is formed by the back-splicing of exon 1 and the upstream 5′ untranslated region of the *Tulp4* gene, with a length of 1649 base pairs (bp). Giusti et al. [29] found that *circTulp4* is a highly expressed circRNA derived from the *Tulp4* gene, enriched in the brain and synaptic regions. Research indicates that *circTulp4* plays a crucial role in regulating neuronal and brain physiology, modulating excitatory neurotransmission, and contributing to neuropsychiatric disorders [30]. Additionally, *circTulp4* is implicated in various diseases. Wu et al. [31] discovered that *circTulp4* regulates β-cell proliferation and may serve as a therapeutic target for type 2 diabetes. Hansen et al. [32] found that *circTULP4* can act as a prognostic marker for biochemical recurrence in prostate cancer. Chen et al. [33] identified its critical biological role in retinal development and degeneration. Han et al. [34] reported that *circTulp4* reduces pyroptosis and alleviates diabetes-related non-alcoholic fatty liver disease by inhibiting the heterogeneous nuclear ribonucleoprotein C/abhydrolase domain-containing 6 axis. *CircSlc8a1* (mmu_circ_0000823) is formed by the back-splicing of exon 1 of the *Slc8a1* gene, with a length of 1830 bp. *CircSlc8a1* plays a key role in neurological diseases and is associated with the neurodegenerative process of Parkinson’s disease [35] as well as the pathogenesis of epilepsy [36]. As a circRNA highly expressed in the heart, it plays a significant role in cardiomyocyte development and homeostasis [37,38] while also contributing to heart diseases such as cardiac hypertrophy [39]. Some studies suggest that *circSLC8A1* may serve as an auxiliary diagnostic marker for sudden cardiac death caused by acute ischemic heart disease [40]. Additionally, *circSLC8A1* is closely linked to cancer, acting as a tumor suppressor in breast cancer, prostate cancer, glioma, non-small cell lung cancer, and bladder cancer. *CircStrn3* (mmu_circ_0000372) is formed by the back-splicing of exon 2 and exon 7 of the *Strn3* gene, with a length of 703 bp. Currently, research on *circStrn3* remains limited. Li et al. [41] found that *circStrn3* regulates osteoarthritis cartilage degeneration by inhibiting chondrocyte matrix metabolism. Gao et al. [42] discovered that *circSTRN3* targets the miR-578/Toll Like Receptor 4 axis, exacerbating sepsis-induced acute kidney injury. Zhang et al. [43] reported that *circStrn3* modulates bone cancer pain in rats. Ling et al. found its association with the pathogenesis of diabetic nephropathy [44]. *CircTulp4*, *circSlc8a1*, and *circStrn3* share high homology with human circular transcripts, making them promising biomarkers for PMI estimation in model establishment.

In this study, mathematical equations were established for low-, room-, and high-temperature environments based on semi-quantitative RT-PCR data, and the corresponding *p*-values, R^2^ values, and error rates were determined. Generally, the closer an equation’s R^2^ value is to 1, the better it fits the data. The *p*-value reflects the reliability of the fitted equation and its ability to represent the data’s characteristics. However, both the sample size and the complexity of the equation can influence the *p*-values, requiring further analysis alongside the validation results of the PMI estimation model. At 4 °C, mathematical equations were successfully constructed by calculating the relative levels of biomarkers to reference genes. Nonlinear equations demonstrated a better fit, with most equations having *p*-values below 0.05. Validation results indicated that *circTulp4* was the most accurate circRNA under the 4 °C conditions. Notably, when combining circRNAs to construct equations, the R^2^ and *p*-values did not change significantly, and the equations using *circCDR1as* as the reference gene exhibited higher accuracy. At 25 °C, the R^2^ values of the mathematical equations were generally high, particularly for cubic equations. However, the *p*-values of cubic equations were often above 0.05, possibly due to the small sample size or random variation. Notably, the R^2^ values improved when circRNAs were combined to construct equations. Among these equations, *circTulp4*/*mt-co1* demonstrated the highest accuracy. In equations combining multiple circRNAs, long-term PMI estimation was more accurate than short-term estimation. At 35 °C, the fit of linear equations was significantly worse than that of nonlinear equations, with *circTulp4*/*28S* rRNA and *circTulp4*/*circSlc8a1*/*circStrn3*/*28S* rRNA models exhibiting poorer fit. Among these equations, those using *circSlc8a1* as a biomarker and the *circTulp4*/*circSlc8a1*/*circStrn3*/*mt-co1* models showed better R^2^ and *p*-values. At 35 °C, nonlinear equations generally exhibited higher accuracy than linear ones, with *circTulp4*/*28S* rRNA equations demonstrating the highest accuracy. The combined application of circRNA mathematical models showed similar accuracy across the three reference genes, and long-term PMI estimation was consistently more accurate than short-term estimation. This study found that combining multiple circRNAs did not significantly improve model accuracy, as the performance primarily depended on the most effective circRNA. However, using multiple circRNAs together can mitigate the impact of differential expression among individual circRNAs, leading to more balanced estimation results. Moreover, the construction of the three-dimensional model enhanced the practical applicability of these circRNAs and offered the potential for PMI estimation under a broader range of temperature conditions. Given the currently limited temperature settings, further exploration of postmortem degradation patterns of circRNAs at additional temperatures will likely improve the accuracy of such models.

This study is the first to utilize a combination of multiple circRNAs from brain tissue for PMI estimation. The research demonstrated that these highly expressed circRNAs in brain tissue also exhibited relatively high expression levels in the heart, skeletal muscle, lung, and kidney, highlighting their potential for combined application across different tissues. The study revealed that PMI and temperature significantly influence the levels of postmortem circRNAs. While experimental conditions can be controlled in the laboratory to eliminate the effects of other factors, real-world environments present greater challenges. Except for temperature, some factors such as microbial activity, pH, and humidity may influence circRNA degradation. In future studies, we plan to incorporate these variables into our PMI estimation models using a weighted approach. By quantifying the influence of each factor and assigning appropriate weights, we hope to enhance the accuracy of the models. Moreover, we anticipate that integrating environmental data (e.g., ambient humidity, pH, microbial load) alongside circRNA expression levels will improve model performance and broaden applicability in varying postmortem contexts. Additionally, the practicality of human circRNA markers corresponding to mouse circular transcripts, such as *circTULP4* (hsa_circ_0131202), *circSLC8A1* (hsa_circ_0005232), and *circSTRN3* (hsa_circ_0031446), requires further investigation due to the lack of study in human samples.

## 4. Materials and Methods

### 4.1. Potential circRNAs and Reference Genes Selection

The human–mouse homologous circRNAs were screened in the literature containing circRNA sequencing data for humans and mice [26,37,45], as well as from existing circRNA databases such as circBase (http://circbase.org/) [46] (accessed on 7 May 2024) and circBank (http://www.circbank.cn/#/home) [47] (accessed on 7 May 2024). Initially, circRNAs exhibiting high expression and conservation in humans and mice were identified from relevant studies. The expression data of corresponding circRNAs were retrieved from circAtlas (https://ngdc.cncb.ac.cn/circatlas/index.php) [48] (accessed on 24 May 2024) and the circBank (accessed on 19 January 2025) database. The circBase IDs of human–mouse homologous circRNAs were then used to gather additional information from circBank, including CircType, SplicedLength, GeneSymbol, and RNA sequences. The identified circular transcripts were further searched in the GTEx (https://www.gtexportal.org/home/index.html) [49] (accessed on 24 May 2024) and NCBI (https://www.ncbi.nlm.nih.gov/gene/) (accessed on 24 May 2024) databases to assess host gene expression across various tissues in humans and mice.

For reference genes, rRNA is often used as a candidate marker for PMI estimation [50,51]. Based on previous studies, the most stable *28S* rRNA was selected as the reference gene [52]. CircRNA CDR1as is a highly abundant circRNA in the mammalian brain, primarily located in the cytoplasm. It is often used as a positive control in circRNA studies related to mammalian brain tissue [11,26,53]. Additionally, cytochrome c oxidase subunit I (*mt-co1*), a highly stable mitochondrial mRNA [54], was also used as a reference gene in this study for constructing the PMI estimation model.

### 4.2. Design of Specific Primers

Primers of circRNAs were designed based on sequences flanking the circRNA back-splicing site. Convergent primers were designed using the linear mRNA sequence corresponding to the circRNA, while divergent primers targeted the back-spliced sequence, which typically connects the 3′ end of the linear transcript with the upstream 5′ end sequence. Primers of circRNAs and reference genes were designed using Primer3 (https://bioinfo.ut.ee/primer3-0.4.0/) (accessed on 17 June 2024) and NCBI BLAST (https://blast.ncbi.nlm.nih.gov/Blast.cgi) (accessed on 17 June 2024), with their specificity verified using the Primer-BLAST tool (https://www.ncbi.nlm.nih.gov/tools/primer-blast/) (accessed on 17 June 2024). The specific divergent primer was further validated using CircPrimer 2.0 software [55] (Center of Clinical Laboratory Science, The Affiliated Cancer Hospital of Nanjing Medical University, Jiangsu, China), which also illustrated their approximate positions within the circRNAs. Detailed primer information is provided in Supplementary Table S8.

### 4.3. Collection of Mouse Brain Tissue Samples

A total of 36 BALB/c male mice (9 and 16 weeks old, weighing 23 to 26 g) were obtained from Tengxin Biotechnology Co., Ltd. (Chongqing, China). The mice were humanely euthanized with intraperitoneal injections of pentobarbital sodium (50 mg/kg) in accordance with AVMA Guidelines for the Euthanasia of Animals (2020 edition) [56] and China’s National Standards “Laboratory Animal-Guidelines for euthanasia (GB/T 39760-2021)” [57]. Brain samples were collected postmortem at different PMIs under varying temperature conditions: 4 °C (0, 1, 2, 4, 8, 12, and 16 days), 25 °C (0, 1, 2, 4, and 8 days), and 35 °C (0, 12, 24, 48, 72, and 96 h). Each PMI group included four samples. Additional brain tissues were extracted at other known PMIs as validation samples, and 11 types of mouse tissues were collected to validate circRNA expressions. All animal experiments complied with relevant ethical guidelines and regulations for laboratory animals in China.

### 4.4. Extraction of Total RNA and Genomic DNA

Total RNA was extracted from approximately 40 mg of brain tissue using the RNAsimple Total RNA Kit (Cat. #: DP210831, Tiangen Biotech Co., Ltd., Beijing, China) following the manufacturer’s protocol. The procedure involved the following steps: 1 mL of Buffer RZ was added to each sample, which was homogenized using a glass homogenizer; the homogenized samples were incubated at room temperature; the samples were centrifuged at 12,000 rpm for 10 min at 4 °C; the supernatant was transferred to a fresh microcentrifuge tube; 200 µL of chloroform was added to the tube, followed by vortexing for 15 s; the mixture was incubated at room temperature for 3 min and then centrifuged at 12,000 rpm for 10 min at 4 °C; the aqueous phase was carefully transferred to a new tube; absolute ethanol (0.5 volume) was added to the aqueous phase; after thorough mixing, the sample was transferred to an RNase-Free Column CR3 placed in a 2 mL RNase-Free Collection Tube and centrifuged at 12,000 rpm for 30 s at 4 °C; the column was washed sequentially with 500 µL Buffer RD and 500 µL Buffer RW, centrifuging at 12,000 rpm for 30 s at 4 °C after each wash; the washing step with Buffer RW was repeated once; the column was returned to the collection tube and centrifuged at 12,000 rpm for 2 min at 4 °C to remove residual ethanol; the RNase-Free Column CR3 was placed in a new 1.5 mL RNase-Free Collection Tube and dried at room temperature for 7 min; and 30 µL of double-distilled H_2_O (ddH_2_O) was added to the column membrane, incubated at room temperature for 2 min, and centrifuged at 12,000 rpm for 2 min at 4 °C to elute the RNA. The concentration and purity of the extracted RNA were measured using a NanoDrop™ 2000 spectrophotometer (Thermo Fisher Scientific Inc., Waltham, MA, USA). RNA purity was deemed acceptable if the A260/A280 ratio was between 1.8 and 2.0 and the A260/A230 ratio was above 2.0. Additionally, 1% agarose gel electrophoresis was performed to assess RNA integrity by visualizing the *5S* rRNA, *18S* rRNA, and *28S* rRNA bands. The extracted RNA was stored at −80 °C or reverse-transcribed into cDNA and stored at −20 °C.

For genomic DNA (gDNA) extraction, the phenol–chloroform method [58] was performed as shown via the following steps: fresh tissue was ground in a 1.5 µL eppendorf (EP) tube using a small grinding stick; a mixture of 700 µL Nucleic Lysis Buffer (0.001 mol/L Tris, 0.04 mol/L NaCl, 0.0002 mol/L EDTA, pH 8.0), 80 µL of 20% SDS (Cat. #: A600848-0100, BBI Life Sciences Corporation, Shanghai, China), and 7 µL of proteinase K (20 mg/mL) (Cat. #: 1245680100, Merck KGaA, Darmstadt, Germany) was added; the mixture was briefly shaken and incubated at 56 °C for 4 h, with vigorous shaking every 30 min; after incubation, an equal volume of Tris-balanced phenol (Cat. #: DP210831, Beijing Solarbio Science & Technology Co., Ltd., Beijing, China) was added, vortexed briefly, and centrifuged at 12,000 rpm for 5 min; the colorless upper aqueous phase was carefully transferred to a new 1.5 µL EP tube; phenol/chloroform (1:1, *V*/*V*) was added, vortexed, and centrifuged at 12,000 rpm for 5 min; the supernatant was transferred to a new tube, and 2.5 volumes of absolute ethanol were added; the white flocculent precipitate was collected by centrifugation at 12,000 rpm for 5 min; the precipitate was washed twice with 70% ethanol, centrifuging at 12,000 rpm for 1 min each time; the DNA pellet was air-dried at room temperature and dissolved in ddH_2_O. The extracted gDNA was stored at −20 °C for subsequent experiments.

### 4.5. Reverse Transcription of Total RNA

Total RNA (1 µg) was reverse transcribed into cDNA using the ReverTra Ace™ qPCR RT Master Mix (Cat. #: FSQ-201, TOYOBO Co., Ltd., Osaka, OS, Japan) in a 10 µL reaction volume containing 5× RT Master Mix, RNA template, and nuclease-free water. The reaction was performed at 37 °C for 15 min, followed by 50 °C for 5 min, and 98 °C for 5 min. The resulting cDNA was stored at –20 °C or diluted tenfold for further experiments.

### 4.6. Validation of Potential circRNAs

Convergent and divergent primers for various circRNAs and the *Actb* control gene are listed in Supplementary Table S8. PCR products were analyzed via agarose gel electrophoresis, where distinct bands from divergent primers confirmed circRNA back-splicing in cDNA. In contrast, no bands were observed with divergent primers in gDNA, validating the circular structure of the circRNAs. For the control gene, amplification was detected only with convergent primers in both cDNA and gDNA.

To further confirm the circular structure of circRNAs, RNase R (Cat. #: R0300; Guangzhou Geneseed Biotech Co., Ltd., Guangzhou, China) digestion was performed. RNase R, an exonuclease derived from *Escherichia coli*, digests linear RNA but not circRNA. Each sample (200 ng of total RNA) was treated with RNase R according to the manufacturer’s protocol. Control samples received reaction buffer only, while treated samples were incubated with RNase R at gradient concentrations (0.25, 0.5, and 1 U). The digestion reaction was carried out at 37 °C for 15 min, followed by enzyme inactivation at 70 °C for 10 min.

Sanger sequencing, the gold standard for circRNA validation, was used to confirm the back-splice junctions of circRNAs. PCR amplification was performed using divergent primers, and the products were sequenced with a 3500Dx Genetic Analyzer (Applied Biosystems, Life Technologies, Carlsbad, CA, USA).

### 4.7. Preparation of the Standard Sample

The standard sample was prepared to minimize the impact of exposure conditions. To generate the standard sample, gDNA from brain tissue was amplified using PCR with mouse *Gapdh* primers. The total reaction volume was 100 μL, consisting of 50 μL of 2× Taq PCR Master Mix (Cat. #: DP210831, Tiangen Biotech Co., Ltd., Beijing, China), 37.5 μL of RNase-free water, 10 μL of primer pairs, and 2.5 μL of gDNA (600 ng). The PCR procedure is detailed in Supplementary Table S9.

### 4.8. Detection of Postmortem circRNA Degradation Levels

The degradation levels of circRNAs across different PMIs at various temperatures were assessed using semi-quantitative RT-PCR. Each reaction had a total volume of 10 μL and contained 5 μL of 2× Taq PCR Master Mix (Cat. #: DP210831, Tiangen Biotech Co., Ltd., Beijing, China), 3 μL of RNase-free water, 1 μL of divergent primer pairs, and 1 μL of cDNA. PCR conditions are detailed in Supplementary Table S9. Amplifications were carried out using Applied Biosystems Veriti^®^ 96-Well Thermal Cycler PCR (Thermo Fisher Scientific Inc., Waltham, MA, USA). The amplified products were separated on a 1.8% agarose gel and visualized using a Bio-Rad Universal Hood II (Bio-Rad Laboratories, Inc., Hercules, CA, USA).

### 4.9. Construction of PMI Estimation Mathematical Models and Validation of the Accuracy

For semi-quantitative RT-PCR analysis, ImageJ 1.54d (National Institutes of Health, Bethesda, MD, USA) was used to measure the integrated density (IntDen) of bands, representing the gray value. To correct for background interference, the background gray value was subtracted from each band’s gray value. Standard samples were included to normalize gray values, ensuring consistency across different agarose gel panels. A fixed volume of the standard sample (3 μL) was loaded onto each gel to enable normalization between circRNA markers and reference genes.

The normalization coefficient (M) was calculated by dividing the background-subtracted gray value of the standard sample in the circRNA agarose panels by that in the reference gene panels. After background correction, the relative expression levels of circRNAs at different PMIs were determined by dividing the average gray value of circRNA markers by that of reference genes at the same PMI. Additionally, a constant (K) was established for the PMI estimation model. The formulas were as follows:

(1) $$K=\frac{M\;\times \;(\mathrm{the}\;\text{background-subtracted}\;\mathrm{gray}\;\mathrm{value}\;\mathrm{of}\;\mathrm{circRNAs}\;\mathrm{at}\;\text{0-day})}{\mathrm{the}\;\text{background-subtracted}\;\mathrm{gray}\;\mathrm{value}\;\mathrm{of}\;\mathrm{reference}\;\mathrm{gene}\;\mathrm{at}\;\text{0-day}}$$

(2) $$\mathrm{Relative}\;\mathrm{levels}\;=\frac{M\;\times \;\left(\mathrm{the}\;\text{background-subtracted}\;\mathrm{gray}\;\mathrm{value}\;\mathrm{of}\;\mathrm{circRNAs}\;\mathrm{at}\;\text{N-PMI}\right)}{\;(\mathrm{the}\;\text{background-subtracted}\;\mathrm{gray}\;\mathrm{value}\;\mathrm{of}\;\mathrm{reference}\;\mathrm{genes}\;\mathrm{at}\;\text{N-PMI})\;\times \;K}$$

where N is the time since death.

To develop the PMI mathematical models, IBM SPSS Statistics 25 (International Business Machines Co., Armonk, NY, USA) was used to construct regression equations and calculate *p*-value and R^2^ for linear and nonlinear models. GraphPad Prism 8 (GraphPad Software, LLC, San Diego, CA, USA) was employed to visualize regression curves for linear, quadratic, and cubic equations.

For model validation, the error rate was used to assess the accuracy of PMI estimation models. The formula was as follows:

(3) $$E\mathrm{rror}\;\mathrm{rate}=\frac{|\;(\mathrm{estimated}\;\mathrm{PMI}\;-\mathrm{real}\;\mathrm{PMI})\;|}{\mathrm{real}\;\mathrm{PMI}}\times \;100\%$$

In addition, based on the relative levels of circRNAs under multiple PMIs at 4 °C, 25 °C, and 35 °C, three-dimensional estimation models were constructed using R software (version 4.2.2, R Foundation for Statistical Computing, Vienna, Austria) packages such as ggplot2, caret, lmtest, and plotly, which may be used to estimate PMI under different temperature conditions.

## 5. Conclusions

In summary, this study developed PMI estimation models for mouse brain tissue under multiple temperature conditions using three highly expressed circRNAs (*circTulp4*, *circSlc8a1*, and *circStrn3*). The findings indicated that *28S* rRNA, *mt-co1*, and *circCDR1as* remain stable across various temperatures, making them suitable reference genes for mathematical models. The combined application of circRNAs demonstrated higher accuracy in long-term PMI estimation. Overall, this study is the first to explore the combined use of multiple circRNAs, potentially offering a novel strategy for PMI estimation. However, further research is needed to assess its practicality for forensic applications.

## Figures and Tables

**Table 1:** Establishment of the mathematical models for 4 °C PMI estimation.

Temperature	circRNA	Reference Gene	Equation		R^2^	*p*
4 °C	*circTulp4*	*circCDR1as*	Linear	Y = −0.02468X + 0.9772	0.7854	0.0014
			Quadratic	Y = 0.9711 − 0.02104X − 0.0002343X^2^	0.7866	0.0118
			Cubic	Y = 1.021 − 0.08117X + 0.009972X^2^ − 0.0004237X^3^	0.8574	0.0080
	*circSlc8a1*	*28S rRNA*	Linear	Y = −0.04908X + 0.9818	0.9132	0.0000
			Quadratic	Y = 1.005 − 0.06299X + 0.0008956X^2^	0.9184	0.0001
			Cubic	Y = 0.9910 − 0.04594X − 0.001997X^2^ + 0.0001201X^3^	0.9201	0.0011
	*circStrn3*	*mt-co1*	Linear	Y = −0.03643X + 0.8845	0.7235	0.0023
			Quadratic	Y = 0.8930 − 0.04148X + 0.0003255X^2^	0.7245	0.0172
			Cubic	Y = 0.9002 − 0.05021X + 0.001806X^2^ − 0.00006147X^3^	0.7251	0.0768
	*circTulp4*/*circSlc8a1*/*circStrn3*	*mt-co1*	Linear	Y = −0.03772X + 0.8588	0.8066	0.0012
			Quadratic	Y = 0.8860 − 0.05393X + 0.001044X^2^	0.8172	0.0081
			Cubic	Y = 0.9411 − 0.1199X + 0.01224X^2^ − 0.0004650X^3^	0.8547	0.0176

**Table 2:** Verification of the 4 °C mathematical models.

Temperature	circRNA	Reference Gene	Real PMI (Day)	Estimation PMI (Day)	Estimation Error (Day)	Error Rate (%)	Equation
4 °C	*circTulp4*	*circCDR1as*	2	1.05	0.95	47.71%	Linear
			2	0.93	1.07	53.64%	Quadratic
			2	0.97	1.03	51.60%	Cubic
			3.125	2.63	0.49	15.83%	Linear
			3.125	2.71	0.41	13.17%	Quadratic
			3.125	1.65	1.47	47.18%	Cubic
			6.25	2.41	3.84	61.40%	Linear
			6.25	2.47	3.78	60.45%	Quadratic
			6.25	1.55	4.70	75.23%	Cubic
	*circSlc8a1*	*28S rRNA*	2	1.40	0.60	30.09%	Linear
			2	1.49	0.51	25.53%	Quadratic
			2	1.59	0.41	20.29%	Cubic
			3.125	4.61	1.48	47.45%	Linear
			3.125	4.21	1.09	34.74%	Quadratic
			3.125	4.48	1.36	43.51%	Cubic
			6.25	10.97	4.72	75.52%	Linear
			6.25	10.48	4.23	67.62%	Quadratic
			6.25	10.17	3.92	62.78%	Cubic
	*circStrn3*	*circCDR1as*	2	2.31	0.31	15.64%	Linear
			2	3.22	1.22	60.84%	Quadratic
			2	0.68	1.32	65.77%	Cubic
			3.125	10.01	6.89	220.37%	Linear
			3.125	11.42	8.29	265.28%	Quadratic
			3.125	10.10	6.97	223.05%	Cubic
			6.25	4.06	2.19	34.99%	Linear
			6.25	6.20	0.05	0.74%	Quadratic
			6.25	6.97	0.72	11.49%	Cubic
	*circTulp4/circSlc8a1/circStrn3*	*circCDR1as*	2	2.93	0.93	46.64%	Linear
			2	3.33	1.33	66.66%	Quadratic
			2	3.91	1.91	95.58%	Cubic
			3.125	6.92	3.80	121.49%	Linear
			3.125	7.85	4.72	151.18%	Quadratic
			3.125	7.83	4.71	150.66%	Cubic
			6.25	6.58	0.33	5.35%	Linear
			6.25	7.51	1.26	20.17%	Quadratic
			6.25	7.55	1.30	20.75%	Cubic

**Table 3:** Establishment of the mathematical models for 25 °C PMI estimation.

Temperature	circRNA	Reference Gene	Equation		R^2^	*p*
25 °C	*circTulp4*	*circCDR1as*	Linear	Y = −0.1129X + 1.051	0.9157	0.0024
			Quadratic	Y = 1.038 − 0.09897X − 0.001686X^2^	0.9167	0.0306
			Cubic	Y = 0.9858 + 0.05610X − 0.06224X^2^ + 0.005272X^3^	0.9392	0.1051
	*circSlc8a1*	*mt-co1*	Linear	Y = −0.1211X + 1.004	0.9299	0.0012
			Quadratic	Y = 1.016 − 0.1335X + 0.001503X^2^	0.9306	0.0194
			Cubic	Y = 0.9814 − 0.03132X − 0.03840X^2^ + 0.003474X^3^	0.9392	0.1292
	*circStrn3*	*circCDR1as*	Linear	Y = −0.1252X + 1.010	0.8933	0.0070
			Quadratic	Y = 1.063 − 0.1818X + 0.006860X^2^	0.9069	0.0499
			Cubic	Y = 0.9836 + 0.05284X − 0.08476X^2^ + 0.007976X^3^	0.9477	0.1074
	*circTulp4/circSlc8a1/circStrn3*	*mt-co1*	Linear	Y = −0.1171X + 1.020	0.9517	0.0011
			Quadratic	Y = 1.011 − 0.1073X − 0.001189X^2^	0.9522	0.0179
			Cubic	Y = 0.9815 − 0.02087X − 0.03496X^2^ + 0.002940X^3^	0.9589	0.1329

**Table 4:** Verification of the 25 °C mathematical models.

Temperature	circRNA	Reference Gene	Real PMI (Day)	Estimation PMI (Day)	Estimation Error (Day)	Error Rate (%)	Equation
25 °C	*circTulp4*	*mt-co1*	2	1.53	0.47	23.30%	Linear
			2	1.86	0.14	7.25%	Quadratic
			2	1.90	0.10	5.20%	Cubic
			6	6.21	0.21	3.49%	Linear
			6	6.49	0.49	8.24%	Quadratic
			6	6.44	0.44	7.39%	Cubic
			7	6.06	0.94	13.42%	Linear
			7	6.39	0.61	8.78%	Quadratic
			7	6.33	0.67	9.53%	Cubic
	*circSlc8a1*	*28S* rRNA	2	5.08	3.08	154.18%	Linear
			2	3.91	1.91	95.58%	Quadratic
			2	3.75	1.75	87.59%	Cubic
			6	7.00	1.00	16.63%	Linear
			6	6.83	0.83	13.89%	Quadratic
			6	5.53	0.47	7.78%	Cubic
			7	6.50	0.50	7.13%	Linear
			7	6.03	0.97	13.87%	Quadratic
			7	8.35	1.35	19.21%	Cubic
	*circStrn3*	*28S* rRNA	2	5.77	3.77	188.43%	Linear
			2	5.17	3.17	158.28%	Quadratic
			2	4.16	2.16	108.07%	Cubic
			6	5.96	0.04	0.68%	Linear
			6	5.40	0.60	10.08%	Quadratic
			6	4.26	1.74	28.97%	Cubic
			7	6.40	0.60	8.55%	Linear
			7	5.97	1.03	14.72%	Quadratic
			7	8.56	1.56	22.29%	Cubic
	*circTulp4/circSlc8a1/circStrn3*	*mt-co1*	2	4.33	2.33	116.38%	Linear
			2	4.42	2.42	121.11%	Quadratic
			2	4.09	2.09	104.26%	Cubic
			6	7.05	1.05	17.50%	Linear
			6	7.06	1.06	17.63%	Quadratic
			6	6.33	0.33	5.47%	Cubic
			7	6.54	0.46	6.54%	Linear
			7	6.58	0.42	6.05%	Quadratic
			7	5.83	1.17	16.68%	Cubic

**Table 5:** Establishment of the mathematical models for 35 °C PMI estimation.

Temperature	circRNA	Reference Gene	Equation		R^2^	*p*
35 °C	*circTulp4*	*mt-co1*	Linear	Y = −0.006651X + 0.8839	0.7999	0.0035
			Quadratic	Y = 0.9293 − 0.01059X + 0.00004125X^2^	0.8209	0.0189
			Cubic	Y = 0.9752 − 0.01976X + 0.0003008X^2^ − 0.000001796X^3^	0.8477	0.0602
	*circSlc8a1*	*mt-co1*	Linear	Y = −0.007844X + 0.7324	0.7186	0.0256
			Quadratic	Y = 0.8961 − 0.02202X + 0.0001486X^2^	0.8943	0.0169
			Cubic	Y = 0.9768 − 0.03815X + 0.0006052X^2^ − 0.000003159X^3^	0.9480	0.0145
	*circStrn3*	*mt-co1*	Linear	Y = −0.006409X + 0.7568	0.6557	0.0341
			Quadratic	Y = 0.8992 − 0.01873X + 0.0001292X^2^	0.8372	0.0260
			Cubic	Y = 0.9582 − 0.03054X + 0.0004632X^2^ − 0.000002311X^3^	0.8765	0.0666
	*circTulp4*/*circSlc8a1*/*circStrn3*	*mt-co1*	Linear	Y = −0.006968X + 0.7911	0.7061	0.0152
			Quadratic	Y = 0.9082 − 0.01711X + 0.0001064X^2^	0.8181	0.0171
			Cubic	Y = 0.9700 − 0.02948X + 0.0004564X^2^ − 0.000002422X^3^	0.8574	0.0321

**Table 6:** Verification of the 35 °C mathematical models.

Temperature	circRNA	Reference Gene	Real PMI (Hour)	Estimation PMI (Hour)	Estimation Error (Hour)	Error Rate (%)	Equation
35 °C	*circTulp4*	*28S* rRNA	24	21.41	2.59	10.80%	Linear
			24	25.19	1.19	4.95%	Quadratic
			24	13.10	10.90	45.41%	Cubic
			30	26.53	3.47	11.57%	Linear
			30	31.74	1.74	5.81%	Quadratic
			30	17.21	12.79	42.62%	Cubic
			60	57.33	2.67	4.45%	Linear
			60	62.70	2.70	4.50%	Quadratic
			60	74.52	14.52	24.21%	Cubic
	*circSlc8a1*	*circCDR1as*	24	51.25	27.25	113.56%	Linear
			24	36.40	12.40	51.69%	Quadratic
			24	30.95	6.95	28.97%	Cubic
			30	33.68	3.68	12.26%	Linear
			30	24.60	5.40	18.01%	Quadratic
			30	19.64	10.36	34.52%	Cubic
			60	70.86	10.86	18.11%	Linear
			60	54.93	5.07	8.44%	Quadratic
			60	76.67	16.67	27.78%	Cubic
	*circStrn3*	*mt-co1*	24	52.73	28.73	119.72%	Linear
			24	33.29	9.29	38.72%	Quadratic
			24	27.67	3.67	15.29%	Cubic
			30	38.53	8.53	28.44%	Linear
			30	25.15	4.85	16.16%	Quadratic
			30	20.30	9.70	32.35%	Cubic
			60	82.38	22.38	37.31%	Linear
			60	64.41	4.41	7.36%	Quadratic
			60	99.49	39.49	65.82%	Cubic
	*circTulp4*/*circSlc8a1*/*circStrn3*	*circCDR1as*	24	46.04	22.04	91.85%	Linear
			24	38.45	14.45	60.19%	Quadratic
			24	35.47	11.47	47.78%	Cubic
			30	38.90	8.90	29.65%	Linear
			30	32.35	2.35	7.82%	Quadratic
			30	28.48	1.52	5.08%	Cubic
			60	71.62	11.62	19.37%	Linear
			60	65.69	5.69	9.49%	Quadratic
			60	74.89	14.89	24.81%	Cubic

**Table 7:** Establishment of the three-dimensional models for PMI estimation.

circRNA	Reference Gene	Equation	R^2^	*p*
*circTulp4*	*28S* rRNA	Y = 0.9424 + 0.0045T + 0.0188P − 0.0001T^2^ − 0.0013P^2^ − 0.0040TP	0.7639	0.0000
	*mt-co1*	Y = 0.6807 + 0.0408T − 0.0297P − 0.0010T^2^ + 0.0011P^2^ − 0.0036TP	0.7998	0.0000
	*circCDR1as*	Y = 0.9195 + 0.0132T − 0.0013P − 0.0003T^2^ − 0.0004P^2^ − 0.0043TP	0.8632	0.0000
*circSlc8a1*	*28S* rRNA	Y = 0.9469 + 0.0220T − 0.0615P − 0.0008T^2^ + 0.0016P^2^ − 0.0031TP	0.8760	0.0000
	*mt-co1*	Y = 0.7840 + 0.0420T − 0.0614P − 0.0013T^2^ + 0.0017P^2^ − 0.0033TP	0.8445	0.0000
	*circCDR1as*	Y = 0.9164 + 0.0257T − 0.0360P − 0.0009T^2^ + 0.0004P^2^ − 0.0040TP	0.8682	0.0000
*circStrn3*	*28S* rRNA	Y = 0.9717 + 0.0069T + 0.0133P − 0.0003T^2^ − 0.0014P^2^ − 0.0045TP	0.7869	0.0000
	*mt-co1*	Y = 0.7959 + 0.0307T − 0.0332P − 0.0009T^2^ + 0.0007P^2^ − 0.0037TP	0.7881	0.0000
	*circCDR1as*	Y = 1.0302 + 0.0041T + 0.0107P − 0.0002T^2^ − 0.0017P^2^ − 0.0046TP	0.8023	0.0000

Note: T: Temperature; P: PMI.

## Data Availability

All data used for the analyses in this report are available from the corresponding author on reasonable request.

## Supplementary Materials

The following supporting information can be downloaded at https://www.mdpi.com/article/10.3390/ijms26104495/s1.
