# Supplementary material for: Development of Mathematical Models Using circRNA Combinations (circTulp4, circSlc8a1, and circStrn3) in Mouse Brain Tissue for Postmortem Interval Estimation
Source: Int J Mol Sci. 2025 May 8;26(10):4495. doi: 10.3390/ijms26104495 (PMC12111416; doi:10.3390/ijms26104495)
Supplement: Supplementary file 1 [file ijms-26-04495-s001.zip › Supplementary figure.pptx]

## Slide 1
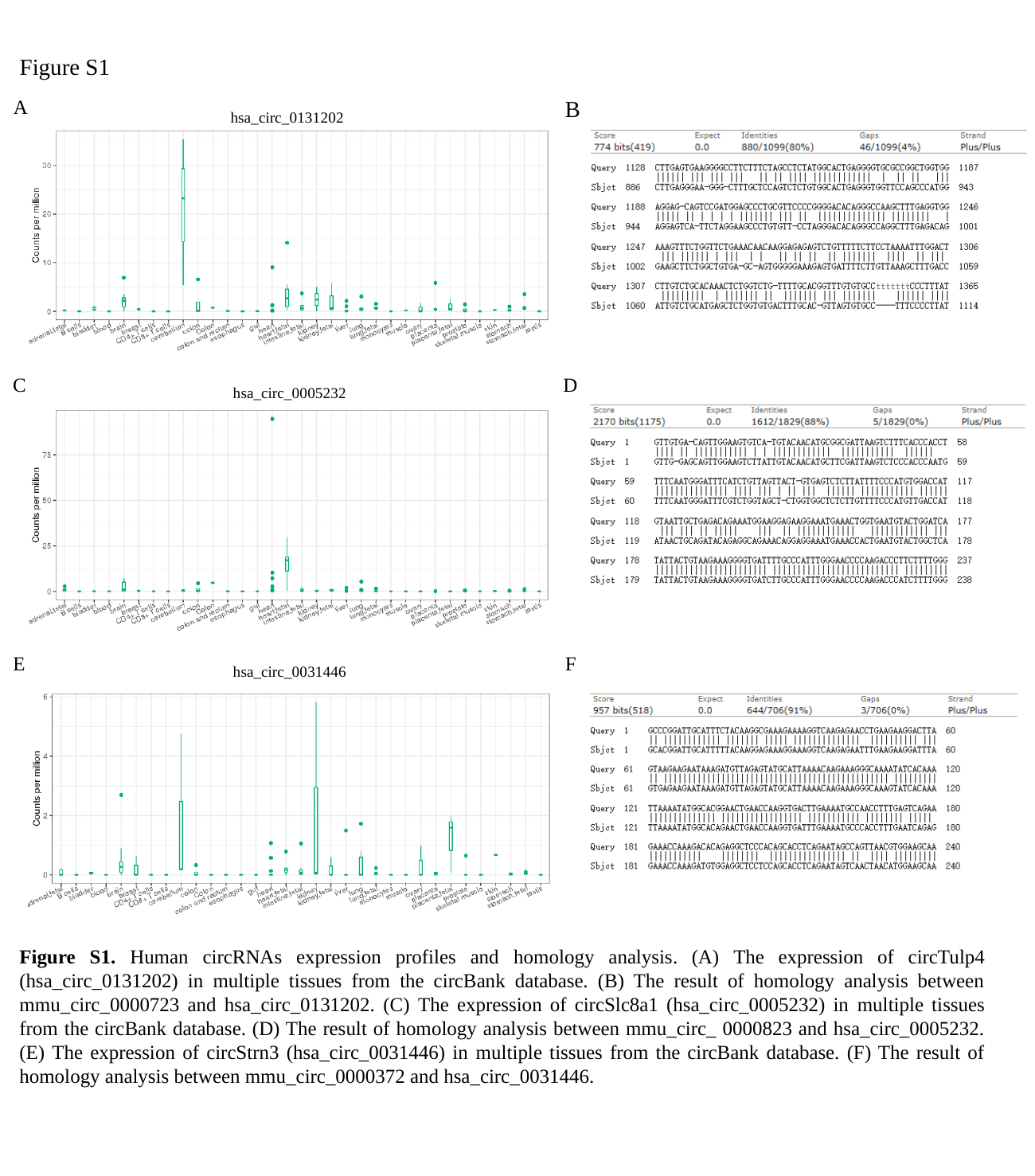

Figure S1
A
B
hsa_circ_0131202
C
D
hsa_circ_0005232
F
E
hsa_circ_0031446
Figure S1. Human circRNAs expression profiles and homology analysis. (A) The expression of circTulp4 (hsa_circ_0131202) in multiple tissues from the circBank database. (B) The result of homology analysis between mmu_circ_0000723 and hsa_circ_0131202. (C) The expression of circSlc8a1 (hsa_circ_0005232) in multiple tissues from the circBank database. (D) The result of homology analysis between mmu_circ_ 0000823 and hsa_circ_0005232. (E) The expression of circStrn3 (hsa_circ_0031446) in multiple tissues from the circBank database. (F) The result of homology analysis between mmu_circ_0000372 and hsa_circ_0031446.

## Slide 2
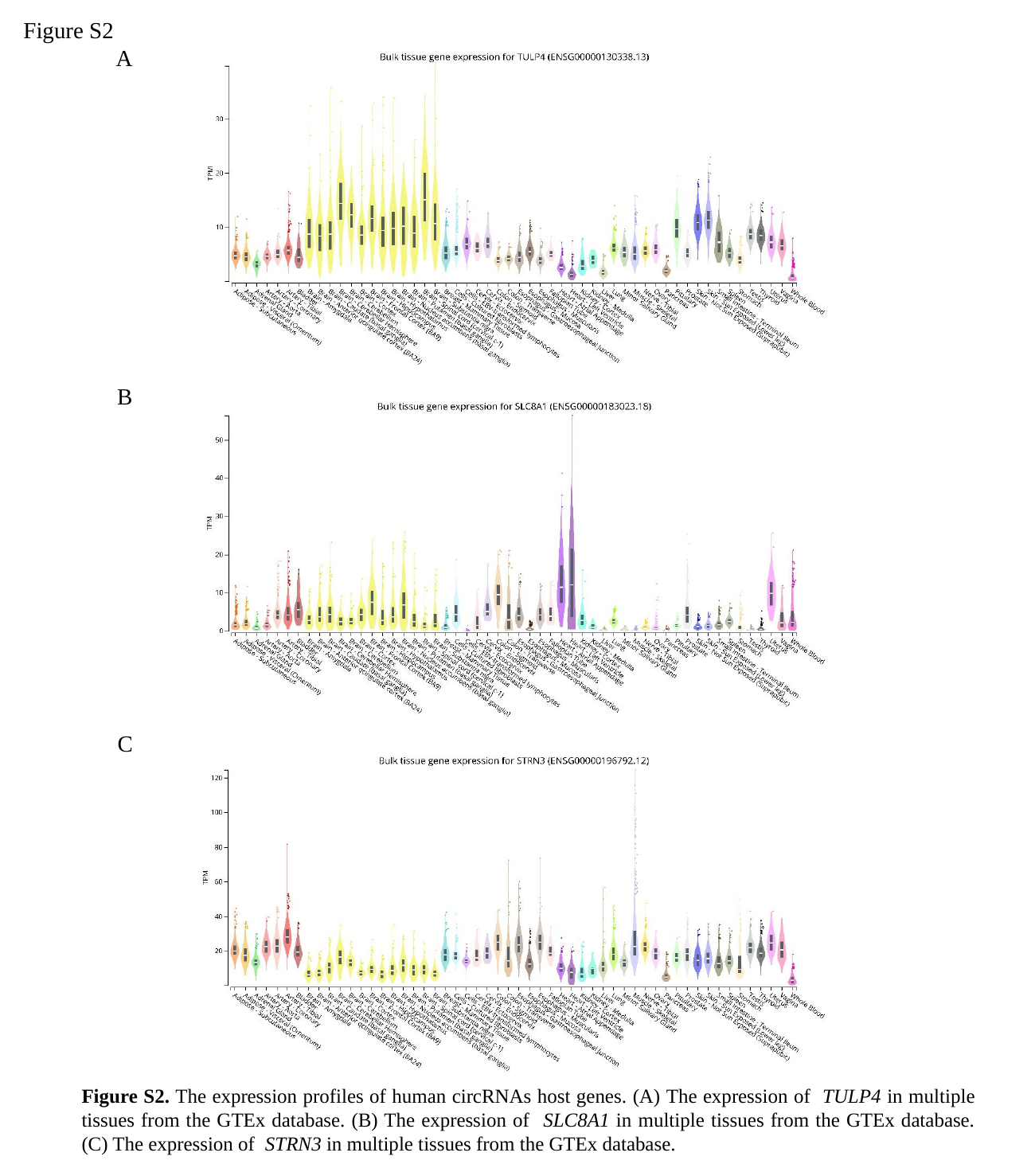

Figure S2
A
B
C
Figure S2. The expression profiles of human circRNAs host genes. (A) The expression of TULP4 in multiple tissues from the GTEx database. (B) The expression of SLC8A1 in multiple tissues from the GTEx database. (C) The expression of STRN3 in multiple tissues from the GTEx database.
